# Air exposure of coral is a significant source of dimethylsulfide (DMS) to the atmosphere

**DOI:** 10.1038/srep36031

**Published:** 2016-10-31

**Authors:** Frances E. Hopkins, Thomas G. Bell, Mingxi Yang, David J. Suggett, Michael Steinke

**Affiliations:** 1 Plymouth Marine Laboratory, Prospect Place, Plymouth, PL1 3DH, United Kingdom; 2 School of Biological Sciences, University of Essex, Wivenhoe Park, Colchester, CO4 3SQ, United Kingdom; 3 Climate Change Research Cluster, University of Technology Sydney, PO Box 123, Broadway NSW 2007, Australia

## Abstract

Corals are prolific producers of dimethylsulfoniopropionate (DMSP). High atmospheric concentrations of the DMSP breakdown product dimethylsulfide (DMS) have been linked to coral reefs during low tides. DMS is a potentially key sulfur source to the tropical atmosphere, but DMS emission from corals during tidal exposure is not well quantified. Here we show that gas phase DMS concentrations (DMS_gas_) increased by an order of magnitude when three Indo-Pacific corals were exposed to air in laboratory experiments. Upon re-submersion, an additional rapid rise in DMS_gas_ was observed, reflecting increased production by the coral and/or dissolution of DMS-rich mucus formed by the coral during air exposure. Depletion in DMS following re-submersion was likely due to biologically-driven conversion of DMS to dimethylsulfoxide (DMSO). Fast Repetition Rate fluorometry showed downregulated photosynthesis during air exposure but rapid recovery upon re-submersion, suggesting that DMS enhances coral tolerance to oxidative stress during a process that can induce photoinhibition. We estimate that DMS emission from exposed coral reefs may be comparable in magnitude to emissions from other marine DMS hotspots. Coral DMS emission likely comprises a regular and significant source of sulfur to the tropical marine atmosphere, which is currently unrecognised in global DMS emission estimates and Earth System Models.

Reef-building corals are prolific producers of dimethylsulfoniopropionate (DMSP), an important precursor to the climate-relevant gas dimethylsulfide, DMS^1^,^2^,^3^. DMSP is produced by both the algal endosymbiont *Symbiodinium*^4^,^5^ and coral host^6^ which, together with the breakdown products DMS and dimethylsulfoxide (DMSO), has many eco-physiological roles in coral reef ecosystems, including infochemistry in organisms from bacteria to fish, and stress protection^7^,^8^,^9^,^10^. Coral-associated DMS production stems from complex interactions in the coral ‘holobiont’ – the combination of coral polyp, endosymbiotic algae and associated microbial community. Importantly, a large proportion of microbes associated with corals are capable of cycling DMSP and DMS, and these compounds play important roles in structuring the bacterial community^6^,^11^. However, there is limited understanding of the mechanisms of DMS production by the coral holobiont and DMS fluxes to the atmosphere from reef environments remain poorly quantified.

Dimethylated sulfur compounds, particularly DMS and DMSO, readily scavenge harmful hydroxyl radicals and other reactive oxygen species^12^, a process which may be beneficial to corals during periods of environmental stress^8^,^10^. High intracellular concentrations of DMSP in *Symbiodinum* sp^5^. coupled with the capacity of DMS and DMSO to diffuse into photosynthetic membranes provides an effective protection mechanism against oxidative stressors^12^. Diffusion across membranes inevitably results in significant release of DMS into the surrounding seawater. Both laboratory and field studies demonstrate strong associations between environmental factors driving oxidative stress (e.g. high light, hyposalinity, high *p*CO_2_) and elevated DMSP, DMS and DMSO in pelagic microalgae^12^,^13^,^14^, benthic macroalgae^15^,^16^,^17^,^18^, and corals^8^,^10^. Recent evidence also demonstrates that DMSP appears to be upregulated by corals during recovery from a thermally-induced bleaching event^19^. At low tide, corals experience high temperature and light levels, air exposure, desiccation and hypoxia^8^. Such conditions are likely to induce stress-related production of DMS(P)-rich mucus by the coral, resulting in elevated DMS concentrations in the over-lying atmosphere^20^,^21^. Indeed, the few observations of atmospheric DMS from reef environments suggest a link between enhanced atmospheric concentrations and low tide events^20^,^22^,^23^. Of particular note are the observations from an atoll in the Florida Keys, where atmospheric DMS increased three orders of magnitude during a tidal cycle - from ~0.01 ppb at high tide up to 19 ppb downwind of the reef at low tide^24^.

Air exposure experiments are a potentially useful tool to evaluate how reef-forming corals contribute to local atmospheric DMS concentrations and regulate dimethylated sulfur compounds during environmental stress. We describe a series of novel laboratory experiments under controlled temperature and light conditions exposing three common Indo-Pacific scleractinian coral species (*Acropora* cf. *horrida, Seriatopora hystrix, Porites cylindrica*) to air for short periods and subsequent re-submersion. Measurements of atmospheric DMS (DMS_gas_), seawater DMS, DMSP, DMSO and Fast Repetition Rate fluorometry (FRRf) were made before, during and after each exposure. Together, our results revealed a marked response in coral sulfur cycling and photophysiological condition that carry important implications both for estimates of sulfur emissions to the tropical/sub-tropical atmosphere and for our understanding of how corals respond to environmental fluctuations.

## Methods

Full details of the analytical methods and coral experimental facility, including coral husbandry, are given in the Supporting Information (SI). Our basic experimental set up was as follows: Independent colonies (genets) of three Indo-Pacific coral species (*Acropora* cf. *horrida, Porites cylindrica n* = *4, Seriatopora hystrix n* = *3*), originally from the same parent colony from the Indo-Pacific, were used to generate between 1 and 25 (mean 11 ± 9) equal-sized nubbins (ramets), 2–5 cm tall, and distributed equally on holding racks receiving one of two light acclimation levels (100 or 400 μmol photons/m^2^/s, indicated by ^100^ and ^400^ throughout the text) for 8–10 weeks before experiments commenced. Ramets were followed throughout experimental design and selected from different genets to ensure biological replication for each treatment. At the beginning of experiments, coral nubbins (ramets) were transferred within a few seconds from the acclimation tank to 3 × 870 mL glass flasks (*n* = 3 or 4 nubbins per flask) with gas-tight lids and fully covered (*submersed*; Stage I) in 500 mL seawater from a 1000 L tropical aquarium. The number of nubbins used was dependent on the number that could be placed inside the flask without the nubbins touching each other or the flask walls. Each flask was positioned within a temperature-controlled water bath at 26 °C illuminated by 54 W fluorescent tubes (Lightwave T5, Growth Technology, UK) delivering ~300 μmol photons/m^2^/s within each flask.

Seawater in the flasks was continuously and gently bubbled with compressed air from tubing inserted to the base of the flask. Set using metering valves and digitally measured (ADM1000, Agilent Technologies), the mean (±SD) flow for all experiments through the flasks was 59.3 ± 6.5 mL/min. It was ensured that neither the tubing nor the bubble stream came into contact with the coral nubbins. Flasks were left for 1 h to approach constant DMS concentrations where net biological production by the coral holobiont was balanced by removal of DMS due to bubbling. Coral nubbins were subsequently exposed to air (*air exposure*; Stage II) by removing seawater from the flasks. The mean exposure time for all experiments (32.7 ± 11.7 min) falls within the range of tidal exposure times of 15–105 min experienced in a natural reef environment^25^. After exposure, flasks were re-filled with the same seawater that had been removed (*re-submersed*; Stage III). When the coral nubbins were (re-)submersed, rising bubbles fully equilibrated with seawater DMS (see Discussion) and exited in the waste air stream. During the air exposure phase the air flow from the base directly displaced the headspace in the flask.

### Gas phase DMS (DMS_gas_) sampling and analysis

**Figure 1:**
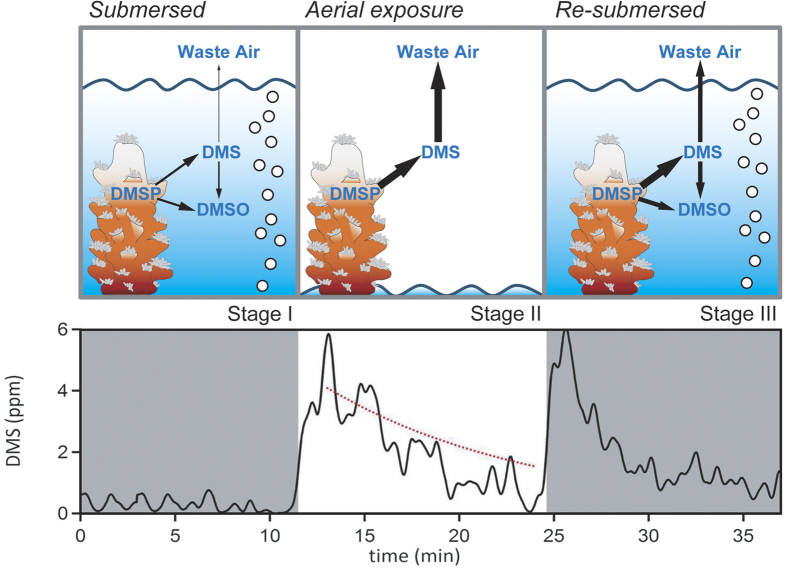
Conceptual diagram of dimethylated sulfur cycling in the presence of coral when *submersed* (Stage I), during *air exposure* (Stage II) and when *re-submersed* (Stage III). Upper panel shows experimental approach with arrow sizes indicating relative strength of production pathways. Lower panel shows high-frequency measurements (black line) of DMS_gas_ in the outflow of a flask containing the coral *Acropora* cf. *horrida*^400^ before, during and after air exposure. The red dashed line shows the calculated loss rate due to flushing of the flask.

**Figure 2:**
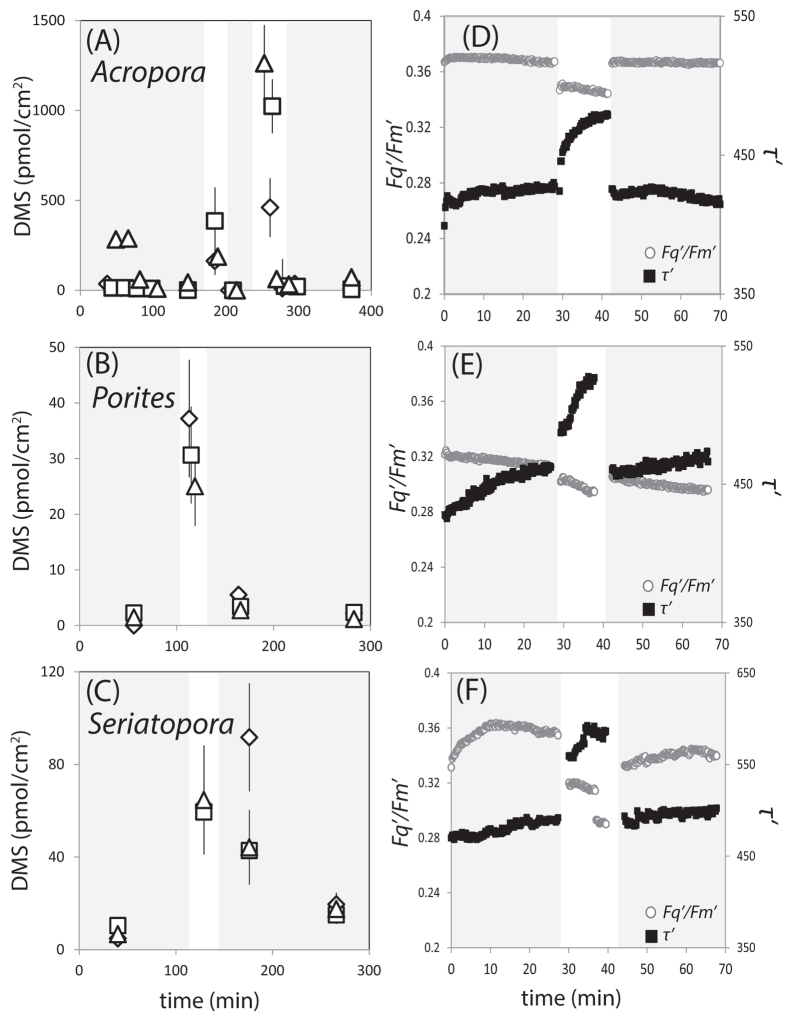
Coral surface area- normalised DMS concentrations (pmol/cm^2^) in the outflow from triplicate coral experimental flasks (**A**–**C**) and photosynthetic performance (**D**–**F**) of coral before, during and after air exposure. Grey shaded areas = coral submersed (Stage I and III), white areas = coral exposed to air (Stage II). *Acropora* cf. *horrida*^400^ (**A**,**D**), *Porites cylindrica*^400^ (**B**,**E**) and *Seriatopora hystrix*^100^ (**C**,**F**). Experiments were run in triplicate and replicate flasks are distinguished by different symbols (Flask 1 = diamonds, Flask 2 = squares, Flask 3 = triangles). Error bars on Panel A represent the total measurement error which includes error associated with 5 min-averaged 0.25 Hz API-CIMS measurements (see Methods), coral surface area measurements and flow rate of culture aeration. Superscript numbers = light acclimation levels in μmol m^−2^ s^−1^. * = discrete measurements made by GC-FPD.

Gas phase DMS concentrations (DMS_gas_) were monitored in the waste air stream from the flasks before, during and after each period of exposure. In experiments with *A.* cf. *horrida*, DMS_gas_ was quantified every 4 s using atmospheric pressure ionisation–chemical ionisation mass spectrometry, API-CIMS (e.g. Fig. 1) (See SI and ref. 23). The other coral species produced DMS at levels that were close to the API-CIMS detection limit (D.L.) (see below). Thus discrete samples from the waste air stream were collected and analysed using the more sensitive, low frequency gas chromatography–flame photometric detection (GC-FPD) method (e.g. Fig. 2B,C)^5^. Similarly, when DMS concentrations from *A.* cf. *horrida* fell below the API-CIMS D.L., discrete samples were taken and analysed via GC-FPD (indicated with asterisks on Fig. 2A).

### API-CIMS

During replicate incubations (Fig. 2), the waste air stream from each flask was connected to an automated selector valve (VICI Valco Instruments Co. Inc.). The valve selected the waste outflow from each flask in sequence (5 min each on three flasks holding corals, 2 min on coral-free control flask). Sample flow from the selected experimental flask was diluted with oxygen-free nitrogen (2 L min^−1^) to make up the total flow required by the API-CIMS instrument^26^. Because pressure needed to be first built up within the flask (after opening) before DMS-laden air was carried out into the API-CIMS, DMS_gas_ measurements from API-CIMS during the first 2 minutes after each stage change (i.e. from submersion to air exposure; from air exposure to re-submersion) are not completely representative of the gas phase DMS within the flasks, and are excluded from analyses. The API-CIMS D.L. within this experimental setup was calculated as 3 standard deviations above the mean of the control ‘no coral’ flasks (0.03 ppm for 5-minute averaged data).

### GC-FPD

Waste gas outflow from experimental flasks was collected for 10 min in 5 L Tedlar bags pre-rinsed with oxygen-free nitrogen (Sigma Aldrich, UK). The bag contents were pre-concentrated on a cryogenic trap at 60 mL min^−1^. The trap was heated rapidly to inject onto the GC column and DMS quantified^5^.

### Water phase DMS, DMSP and DMSO

In a separate experiment without bubbling with compressed air, seawater DMS, total aqueous phase DMSP (DMSP_t_) and dissolved DMSO (DMSO_d_) samples were taken before and after coral exposure. For DMS, 5 mL samples of pre-filtered seawater media were analysed via purge and cryogenic trap followed by GC-FPD^27^. DMSP in seawater media was determined via cold alkaline hydrolysis to DMS, and purge and cryogenic trap followed by GC-FPD^5^,^28^. DMSO concentrations were determined via reduction to DMS following the addition of NaBH_4_ to seawater samples, and purge and cryogenic trap followed by GC-FPD^29^,^30^.

### Coral photophysiology

Fast Repetition Rate fluorometry (FRRf; *Fasttracka II*, Chelsea Technologies Limited, UK) data was collected from coral nubbins during parallel air exposure experiments. FRRf inductions (0.2 Hz) parameterised the photosynthetic efficiency (*F*_*q*_´/*F*_*m*_´, dimensionless), effective absorption cross sectional area (σ´, nm^2^) and electron turnover time (τ´, μs)^31^.

## Results

### Stage I: Initial coral submersion

During initial submersion, DMS_gas_ for *A.* cf. *horrida*^400^ ranged from 0.2–0.8 ppm (1.2–288 pmol/cm^2^), whilst the other species were within a lower range of 0.05–0.2 ppm (0.01-10.4 pmol/cm^2^) (Figs 1 and 2A–C, and Table 1). It is important to note that these observations should not be directly compared to ambient DMS mixing ratios in the marine atmosphere, which are subject to various processes including different degrees of dilution and photochemical degradation. However, the higher DMS_gas_ in *A.* cf. *horrida*^400^ experiments is qualitatively consistent with the higher DMSP content for that species. Specifically, DMSP content (normalised by the coral surface area) was 900–1600 nmol/cm^2^ for *A.* cf. *horrida* but 40–80 nmol/cm^2^ for the remaining three species (see SI and Table S1). Our *A.* cf*. horrida* DMSP content is well within the range of reported values for other *Acropora* spp. (34–3558 nmol DMSP/cm^2^)^1^,^3^,^8^,^19^,^32^.

### Stage II: air exposure of corals

High temporal resolution API-CIMS measurements of DMS_gas_ from a flask containing a colony of *A.* cf. *horrida* increased by about an order of magnitude from 0.3 ± 0.2 ppm (mean ± SE) during submersion up to a maximum of 5.8 ppm within the first 2 minutes after air exposure (Fig. 1).

This initial spike in DMS_gas_ during air exposure is expected since DMS is now directly emitted into the gas phase rather than first into the water phase. DMS is very soluble - the dimensionless solubility of DMS (water phase concentration:gas phase concentration) is 11.4 at 26 °C^33^. Thus at equilibrium during (re-)submersion, the surrounding seawater acts as a DMS ‘reservoir’ with ca. 95% of the DMS by mole residing in the water phase at a given time. When the water is removed, DMS produced by the corals is directly emitted into the gas phase, resulting in high DMS_gas_ within the first minutes after air exposure. After the initial spike, DMS_gas_ steadily decreased during the subsequent 12 minutes and returned to near baseline levels. We estimate the exponential decay in DMS_gas_ due to flushing with DMS-free compressed air (red dashed line in Fig. 1). Air dilution can account for essentially all of the DMS_gas_ decrease during minutes 12 to 24, which suggests that after the first few minutes of air exposure, there is very limited additional DMS emission from the corals (which would cause observed DMS_gas_ to be above the modeled air dilution line). We note that this observation does not imply zero DMS production from the coral holobiont upon air exposure. DMS production likely continues leading to the initial spike in DMS_gas_, but is then trapped by the mucus layer that quickly forms on the coral surface upon exposure (see Discussion).

We repeated the experiment with nubbins of *A.* cf. *horrida* (Fig. 2A), and with nubbins of two further species: *Porites cylindrica*^400^ (Fig. 2B) and *Seriatopora hystrix*^100^ (Fig. 2C). Air exposure of all species resulted in about an order of magnitude increase in DMS_gas_. DMS_gas_ averaged from the first 10 minutes of air exposure varied among the different coral species: *A.* cf. *horrida* gave the highest value of 3.4 ± 0.3 ppm (1262 ± 213 pmol/cm^2^), while the other species yielded less than 1 ppm (<100 pmol/cm^2^). Both raw DMS_gas_ concentrations and zooxanthellae cell density-normalised DMS concentrations yielded the same trends (Table 1 and Figure S1). Again, this may be a reflection of the variability in tissue DMSP content between the tested species (Table S1). It is worth noting that we did not perform our experiments at light levels likely to be encountered in the reef environment (700–1200 μmol photons/m^2^/s)^34^ to see whether or not DMS production is further enhanced under these conditions. However, we did perform a further experiment in the dark with two flasks holding *A.* cf. *horrida*^100^ and two flasks holding *A.* cf. *horrida*^*4*00^ (*n* = 3 nubbins per flask). The same response was seen during air exposure, with DMS_gas_ reaching maxima that ranged from 0.8–4 ppm across four experimental flasks, compared to 0.1–1.5 ppm during submersion (Figure S2). No significant differences in DMS_gas_ were detectable between *A.* cf. *horrida*^100^ and *A.* cf. *horrida*^*4*00^.

To convert the gas phase mixing ratios above to DMS emissions (i.e. flux) from the coral, we started from the mass balance equation of gas phase DMS:


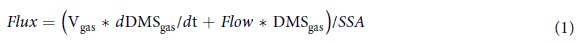


Here the headspace volume during air exposure V_gas_ = 700 mL, the gas flow *Flow* = 60 mL/min. To make the coral emissions comparable to other marine DMS flux hotspots, we converted our flux estimates into equivalent sea surface areas (*SSA*); we take *SSA* for each fragment in our experiments to be the birds-eye-view water surface area above the coral (estimated as 25 cm^2^ for each fragment x 3 nubbins = 75 cm^2^). Eq. 1 requires the observed rate of change in DMS_gas_ increase (*d*DMS_gas_/*d*t), which we could not accurately quantify due to the measurement system design. For simplicity, we therefore assume the averaged DMS_gas_ shown in Fig. 2 to be the peak values upon air exposure, which were achieved within two minutes. Using this approach, the coral-to-air emission of *A.* cf. *horrida* was 3–11 mmol/m^2^/day. Emissions from other coral species were lower, at 1.7 mmol/m^2^/day for *P. cylindrica* and 2.3 mmol/m^2^/day for *S. hystrix.*

### Stage III: coral re-submersion

Re-submersion of *A.* cf. *horrida* at 24 minutes into the experiment resulted in a second rapid increase of DMS_gas_ up to 6 ppm in the outflow of the flask, followed by a near-exponential decrease in concentration (Fig. 1). Unlike the transition from submersion to air exposure, the initially high DMS_gas_ after re-submersion is opposite to the expected solubility-driven response when moving from air to water. Instead, our observations imply high concentrations of DMS in the seawater when the coral is first re-submersed. This could be due to rapid production of DMS by the coral holobiont. Alternatively, if substantial DMS was trapped in the mucus during air exposure (Stage II), re-submersing the coral in seawater could result in dissolution and liberation of the DMS trapped in mucus. The subsequent decrease in DMS_gas_ is driven by biological processes associated with the presence of coral, with water-to-air emission accounting for only a quarter of the DMS loss (see Discussion).

### Coral photophysiology during submersion, air exposure, and re-submersion

Continuous FRRf measurements provided a diagnostic of photosynthetic performance before, during, and after air exposure. Photosynthetic downregulation was identified by a temporary decrease in the effective absorption cross-section (σ´) (Table S2) and the operating efficiency of photosystem II (*F*_*q*_*’/F*_*m*_’) in conjunction with an increased photosynthetic electron turnover time (τ´) (Fig. 2D–F). This is in contrast to photo-damage by reactive oxygen species that results in a sustained increase in σ´ and/or τ´ coupled with a decrease in *F*_*q*_*’/F*_*m*_’^35^,^36^. The FRRf-derived response of *A.* cf. *horrida*^400^ indicated that photosynthesis downregulated during air exposure but consistently recovered upon re-submersion, suggesting no prolonged down-regulation that would result from photo-damage (Fig. 2D). *P. cylindrica*^400^ and *S. hystrix*^100^ responded similarly to *A.* cf. *horrida*^400^, but showed less recovery in *F*_*q*_*’/F*_*m*_’, which remained below the level measured before air exposure (Fig. 2E,F, Table S2). The full recovery of *F*_*q*_*’/F*_*m*_’ by *A.* cf. *horrida* suggests a greater tolerance to the physiological stress of transient air exposure compared to the other species tested here. *Acroporids* are well reported to thrive in intertidal zones that experience prolonged (>3.5 h) periods of exposure during spring tides^37^. Furthermore, there is evidence that specimens adapted to such variable environments show greater physiological tolerance to thermal stress^38^,^39^. Thus *Acroporids* may possess physiological mechanisms that instil tolerance to a range of environmental conditions.

### Seawater sulfur dynamics after coral re-submersion

**Figure 3:**
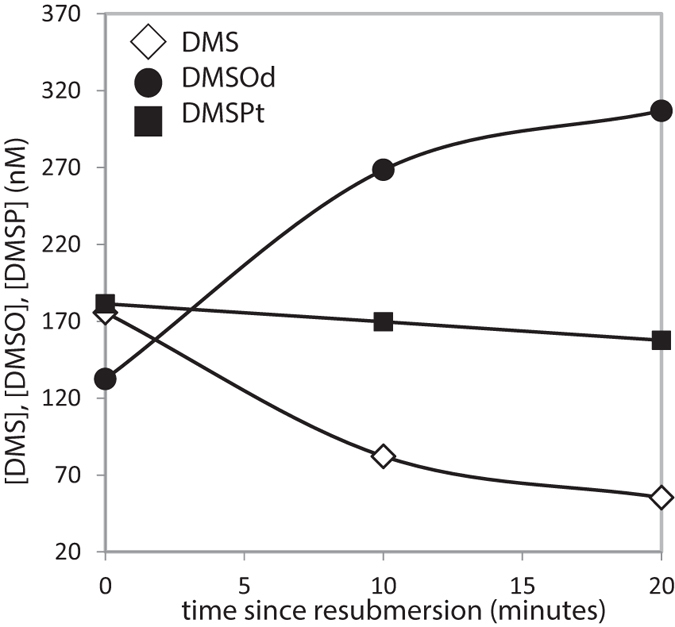
Seawater concentrations of DMS, total DMSP (DMSP_t_) and dissolved DMSO (DMSO_d_) (nM) in a sealed flask containing nubbins of *A.* cf. *horrida*^400^. Single samples were taken at 10 min intervals following re-submersion (Stage III) of the coral after a short (15 min) period of air exposure. The error on the measurements (±1.1 nM) falls within the size of the symbols.

To explore the roles of dimethylated sulfur compounds as anti-oxidants, we measured the seawater concentrations of DMS, dissolved DMSO (DMSO_d_) and total aqueous phase DMSP (DMSP_t_) at regular intervals after re-submersion of *A.* cf. *horrida*^400^ in a separate experiment (Fig. 3). Here, 3 x *A.* cf. *horrida* nubbins were transferred to a single flask and subjected to a 15 min air exposure, before the flask was re-filled with seawater. In contrast to all other experiments, the flask was not flushed with air (i.e. minimal water-to-air emission of DMS). Abiotic photochemical loss of DMS, inferred from previous measurements from the Sargasso Sea^40^, is also slow (at most 0.006/h). Rapid changes occurred during the first 10 minutes, corresponding to a rate constant for net DMS loss of about 3.7/h, which we can largely attribute to coral-related biological processes as follows:

After 20 minutes, DMS levels had decreased by 120 nM (equivalent to 1.0 nmol/cm^2^, based on the mean surface area (41.9 ± 27.8 cm^2^ per nubbin, see Table S3) of 3 x *A.* cf. *horrida* fragments). Simultaneously, DMSO_d_ increased by 175 nM (1.4 nmol/cm^2^). We see that the net increase in DMSO concentration (the principal oxidation product of DMS) was 55 nM higher than the net decrease in DMS concentration. Using a simplistic mass balance where no DMSO is directly released by the coral, 100% of DMS is converted to DMSO, and DMSO loss is negligible, Fig. 3 suggests a gross DMS production of 55 nM (175–120 nM) and a gross DMS consumption (converted to DMSO) of 175 nM over the 20 minutes. Within the first 10 minutes after re-submersion when the rates were the highest, this corresponds to a gross DMS production rate of 300 nM/h and a gross DMS consumption rate of 840 nM/h. Of course, less than unity conversion from DMS to DMSO is likely^41^, as well as significant consumption of DMSO^42^,^43^, which together would imply greater DMS gross consumption. On the other hand, any direct coral emission of DMSO^8^ would reduce the DMS gross consumption required for mass balance (at the limit of no DMS production, DMS gross consumption = DMS net consumption = 540 nM/hr within the first 10 minutes).

Interestingly, DMSP_t_ levels in seawater did not change significantly, suggesting fairly low *in situ* DMSPt to DMS conversion in the seawater. It is worth noting that this seawater DMSP_t_ concentration is orders of magnitude lower than the DMSP content in corals. Together, these lines of evidence suggest that DMS emitted after re-submersion of *A.* cf. *horrida* is rapidly turned over, likely in large part to DMSO.

## Discussion

**Figure 4:**
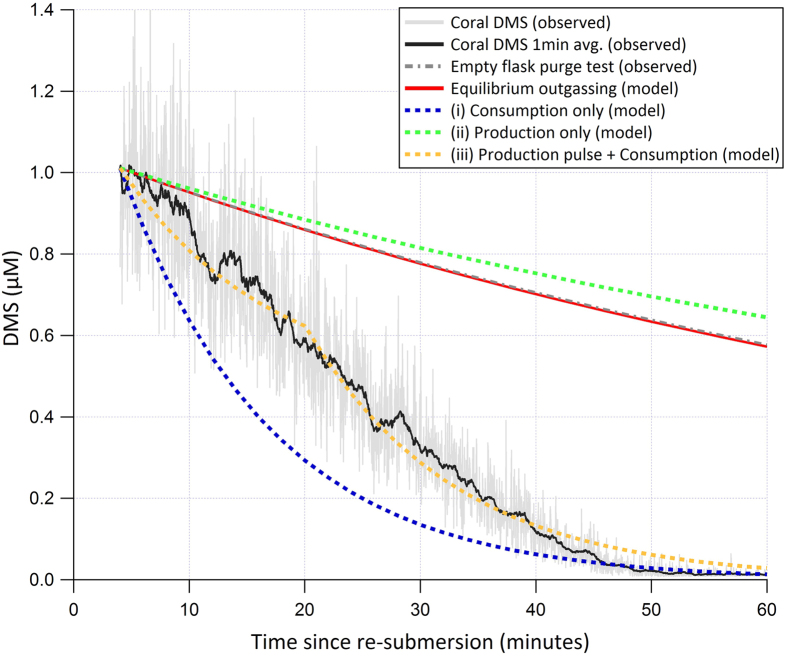
Observations and model results for DMS_sw_ in an experimental flask holding an *Acropora* cf. *horrida* colony. API-CIMS measurements (black line: 1 min average) commenced upon coral re-submersion following 16 min of exposure to air (Stage III). Grey dashed line represents the exponential loss of DMS due to bubbling based on measurements made in a coral-free flask derived during a separate experiment. The red line shows the calculated loss rate due to equilibrium outgassing. Results of model runs: (i) loss rate in absence of DMS production with a first order consumption rate of 3.7/hr (blue dashed line); (ii) zero consumption and a constant production rate of 100 nM/h (green dashed line); (iii) first order consumption rate of 3.7/hr and high production of 2 μM/h for first 15 min followed by zero production (gold dashed line).

We developed a simple box model in order to explore the plausible bounds of biological DMS production and consumption. As an independent measure, we used the biological consumption rate derived from the no-bubbling experiment (Fig. 3) to model the decay in DMS a few minutes after re-submersion of *A.* cf. *horrida*^400^ nubbins (*n* = 3 in a single flask) in a separate experiment (Fig. 4). The coral nubbins had been subjected to 15 minutes of air exposure before re-submersion and the airside mixing ratio was measured at a high temporal resolution by API-CIMS over an hour (Fig. 4). DMS in the gas phase is expected to be at equilibrium with the water phase during re-submersion. The equations from^44^ predict that in our experimental setup (bubble radius <1 mm, water level height = 20 cm) bubbles should be fully equilibrated with the surrounding DMS_sw_ when they surface. We verified this by adding aqueous DMS standard to 500 mL of coral-free seawater (final concentration = 100 nM) and monitored the rate of decrease due to bubbling (60 mL/min) in a separate test. The observed reduction (grey dash-dotted line in Fig. 4) in DMS_gas_ from bubbling fit to an exponential decay: *% loss* = *100.exp*^*−0.01t*^ where *t* = time in minutes (R^2^ = 0.9), which shows excellent agreement with the modelled DMS equilibrium outgassing (red line). Based on this, we convert the API-CIMS gas phase DMS mixing ratio to a waterside concentration (DMS_sw_, in units of μM) via the ideal gas law and the Henry’s solubility.

Our box model (similar to^45^) consists of a time-dependent representation of DMS outgassing at equilibrium rate (water-gas transfer) as well as biological DMS production and consumption. DMS consumption is modelled as a first order process: seawater DMS concentration (e.g. in units of nM) multiplied by the rate constant for DMS consumption (e.g. in units of 1/hr). DMS production (e.g. in units of nM/hr) is assumed to be independent of the DMS concentration. We initialised the model with the observed DMS_sw_ a few minutes after re-submersion. Similar to previous experiments, elevated DMS_sw_ (~1 μM) was observed immediately after the coral was re-submersed followed by a gradual decline in concentration to ~0.01 μM after 60 minutes. The model shows that equilibrium outgassing can only explain about 25% of the total DMS loss within the first 20 minutes after re-submersion. This indicates additional coral-associated loss of DMS_sw_, similar to Fig. 3. When the model is run with just production (100 nM/hr), gas exchange, and zero biological consumption (green dashed line in Fig. 4), the model overestimates DMS_sw_ relative to observations. A consumption-only model run (rate constant of 3.7/hr, with gas exchange but no production; blue dashed line in Fig. 4) significantly underestimates. We chose 3.7/hr because it is the rate constant for net consumption in Fig. 3 over the first 10 minutes after re-submersion. A best fit to the observations in Fig. 4 was achieved with a 15 minute pulse of high DMS production (2 μM/hr, gold dashed line) at the beginning of the experiment, before turning off DMS production, accompanied by constant consumption rate constant of 3.7/hr.

An increase in DMS production by the coral holobiont upon re-submersion could have been partly driven by increased bacterial DMS production or increased enzymatic cleavage of DMSP to DMS by the symbiotic algae. Compromised endosymbiotic algae and/or the coral polyp can enhance the availability of dissolved DMSP through leakage into the surrounding seawater, which in turn would increase bacterial DMS production. However, our FRRf data do not suggest compromised endosymbionts and so DMSP_d_ leakage from the algae was probably minimal. This would also be consistent with the fairly constant seawater DMSP_t_ concentration measured.

Direct DMS production by algae is recognised, particularly under conditions of environmental stress^46^. As such, enzymatic conversion of DMSP to DMS by the symbiotic algae would provide antioxidants close to the photosynthetic apparatus where oxidative stress is high. Alternatively, a stress-induced increase in *de novo* DMSP production by the symbiotic algae during air exposure may stimulate an increase in bacterial DMSP cleavage to DMS within the mucus layer^21^. Our model results give a best fit to the re-submersion data when there is a pulse of high DMS production after re-submersion (Fig. 4). Such short-term up-regulation of DMS production would peak when stress levels are likely to be at their maximum.

Re-submersion of corals after air exposure re-oxygenates the tissues, causing stress through the over-production of reactive oxygen species^47^,^48^. Sea anemones and sea pens, close relatives of scleractinian corals, display a pre-emptive defence strategy during air exposure that includes the activation of antioxidant mechanisms, such as upregulation of heat shock protein (HSP), catalase (CAT) and/or glutathione-S-transferase (GST), to minimise the free radical damage associated with re-oxygenation^47^,^48^. DMS reacts rapidly with reactive oxygen species (ROS) such as the hydroxyl radical (•OH) to form, in particular, DMSO^12^. The rapid reduction in DMS concentrations and concurrent increase in DMSO after re-submersion (Fig. 3) could therefore be driven by *in vivo* ROS-scavenging reactions. Recent work implicates a DMSP-based antioxidant system in the ability of the closely-related species *Acropora millepora* to withstand hyposalinity stress^10^. Our observations of dimethylated sulfur compounds and photophysiology support a further antioxidant role for DMS(P) production by *A.* cf. *horrida* as a mechanism to cope with rapid re-oxygenation and oxidative stress.

The rapid turnover of DMS to DMSO could also be an incidental antioxidant reaction, resulting from the presence of coral but not directly controlled by the coral. ROS such as H_2_O_2_ are produced by both photosynthetic symbionts^36^ and the coral animals^49^, and are released into surrounding seawater by diffusion or active water exchange^50^. The role of mucus in H_2_O_2_ release is unclear; a previous study reports undetectable levels in mucus from *Stylophora pistillata*^49^. However, rapid H_2_O_2_ release by *S. pistillata* has been reported to be stimulated by both chemical and physical stressors^49^. The physical and chemical stimuli associated with re-submersion following air exposure could result in a burst of H_2_O_2_ production. DMSO is the first product of the aqueous phase reaction between DMS and H_2_O_2_^51^,^52^, so coincident release of H_2_O_2_ could result in rapid oxidation of dissolved DMS to DMSO.

It is worth mentioning that even a high DMS production of 2 μM/h (see Fig. 4) does not come close to explaining the initial spike in DMS_sw_ after re-submersion. A net DMS production of 15 μM/h would be required to achieve ~1 μM after only four minutes. We noticed that coral re-submersion caused the release of mucus ropes, which typically contain DMS(P) concentrations that are orders of magnitude higher than in seawater^2^. During air exposure, bio-physical mechanisms such as mucus production^53^ and/or polyp retraction^54^ help to minimise water loss but also limit the cutaneous oxygen supply and potentially cause hypoxia within the tissues^53^. After the first few minutes of air exposure, we did not detect much additional DMS emission so these mechanisms may also restrict the release of DMS when coral is exposed to air, thereby possibly allowing DMS to build up within the mucus. The dissolution of this DMS-rich mucus into the surrounding seawater may contribute to the observed pulse of DMS at the point of re-submersion. We measured the concentration of DMS in mucus and mucus ropes produced by a colony of *A.* cf. *horrida* that had been exposed to air for 15 minutes (a separate colony to those used in the main experiments - see SI methods). Concentrations ranged from 279–577 nM in mucus, with a value of 373 nM in a sample of mucus rope (interestingly, mucus similarly collected from *P. cylindrica* gave undetectable levels of DMS (D.L ≤50 nM, see ref. 5)). Our values for *A.* cf. *horrida* fall well within the range of values previously reported for other Acroporids (61–18665 nM)^2^.

Using this information, we can estimate the resultant [DMS_sw_] upon dissolution of DMS-rich mucus into the water phase:


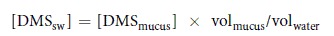


Mucus thickness after 15 min of exposure is estimated to be 0.05 cm, based on a range of 0.01 to 0.1 cm reported for *A. millepora*^55^. The mean surface area of 3 x *A.* cf. *horrida* nubbins used in our study is 125.7 cm^2^ (See Table S3). Assuming the entire coral fragment develops an even coating of mucus, this gives a volume of mucus (vol_mucus_) of 0.006 L. Seawater volume in the flask (vol_water_) was 0.5 L. Taking the maximum value of [DMS_mucus_] we measured for *A.* cf. *horrida* (577 nM) would result in [DMS_sw_] of only 7.3 nM, far from the ~1 μM we measured (Fig. 4). Using the maximum reported value from the literature of 18665 nM would result in [DMS_sw_] of 235 nM. This implies that the spike in DMS concentrations upon submersion is not simply a result of the dissolution of DMS-rich mucus, and instead may be driven by a short-term up-regulation in DMS production by the coral holobiont.

Our experiments suggest that coral reefs represent an as-yet unquantified all-year-round direct DMS flux into the tropical atmosphere (~23°N to 23°S), which is not currently accounted for in existing climatologies and Earth System models^56^,^57^,^58^. The coral-to-air flux of DMS (per sea surface area occupied by the coral) from *A.* cf. *horrida* was 3–11 mmole/m^2^/day, with lower emissions from other coral species including *P. cylindrica* and *S. hystrix*. To put this emission in environmental context requires knowledge of the areal extent and species composition of exposed reef as well as the frequency of exposure (tidal cycle and reef height). The extent of exposed coral reef during low tide events is not well documented but typically includes the reef crest^59^. Frequency of coral exposure is also poorly quantified. As a case study, we use the observations from Heron Island, a well-studied reef at the southern tip of the Great Barrier Reef. The circular reef rim has 15.8% coverage by *Acropora* colonies and is exposed on average 12 hours per month (~0.5 days per 30 days = ~0.02)^25^,^54^. From this, we estimate the long-term average DMS flux from the reef rim as 9–35 μmol/m^2^/day (i.e. 3–11 mmol/m^2^/day *(0.158)*0.02*1000) by assuming that *Acropora* on the reef rim are the sole source of DMS and only emit to the atmosphere when exposed to air. This simplistic calculation does not account for the complexity of the reef environment (e.g. the variability in time of exposure that different areas of the reef experience, or the contributions from non-*Acropora* corals and other symbiotic cnidarians). Furthermore, the experimental exposure times used here (32.7 ± 11.7 min) fall short of maximum exposure times in a natural reef environment (up to >100 min)^25^. A longer exposure time may result in further deoxygenation of tissues, which upon re-submersion and re-oxygenation^47^,^48^ may be compensated for by an enhanced pulse of DMS production. However, without further data, predicting the effects of increased exposure time on the DMS flux is highly speculative. Thus, our uncertainty in this flux estimate is large and future observations will need to overcome these knowledge gaps to reconcile and scale natural from laboratory-derived fluxes.

Nevertheless, DMS emissions from corals may be in the same order of magnitude as other marine DMS hot spots, which tend to be more transient. For example, DMSP-rich surface pack ice in Antarctica is estimated to emit DMS to the atmosphere at a few tens of μmol/m^2^/day^60^. Strong open ocean sea-to-air DMS flux up to 100 μmol/m^2^/day were measured over a coccolithophore-dominated bloom in the North Atlantic^61^, whilst a flux of 30 μmol/m^2^/day was seen over a dinoflagellate bloom in the Southern Ocean^62^. Atmospheric DMS was generally less than <1 ppb over these algal blooms - significantly lower than the mixing ratio of 19 ppb that have been observed downwind of coral reefs^24^. Phytoplankton blooms typically last only days to weeks, with maximum DMS production persisting for a relatively short period, often during the senescent phase^63^. In contrast, tidal exposure of coral reefs may provide a strong regular DMS flux to the atmosphere.

Our experiments show that when water is removed and corals are exposed to air, a solubility effect results in an order of magnitude increase in DMS concentrations in the overlying air in the first few minutes after exposure. This is consistent with observations of elevated DMS in the air above coral reefs following low tides^22^,^23^,^24^. Re-submersion of coral resulted in another short-lived pulse of DMS, which might be due to a combination of greater DMS production by the coral holobiont and/or dissolution of DMS-rich mucus that may be formed during air exposure. At low tide, corals inhabiting reef flats and crests can experience significant periods of air exposure, subjecting them to thermal stress, high irradiance and desiccation^64^,^65^. We propose that DMS production by corals may help to counter the stress associated with cycles of tidal exposure and re-submersion with rapid reoxygenation of tissues. Our data suggest that tidally exposed coral reef rims should be considered important DMS hotspots, in particular where species of *Acropora* dominate coral communities. *Acropora* is a highly successful genus contributing to rich species diversity and productivity on coral reefs via their complex architecture and rapid growth^66^. *Acropora* is also particularly susceptible to major environmental and climatic stressors, which are expected to increase in frequency in the future^67^. Significant changes in *Acropora* cover could impact upon future biodiversity and productivity associated with coral reefs^66^. Such changes in coral speciation would also have implications for local atmospheric fluxes of DMS^68^. *In situ* measurements of atmospheric DMS concentrations and coral-to-air DMS fluxes in reef environments at a range of temporal and spatial scales are required to better quantify the importance of DMS in the tropical marine atmospheric chemistry.

## Additional Information

**How to cite this article**: Hopkins, F. E. *et al*. Air exposure of coral is a significant source of dimethylsulfide (DMS) to the atmosphere. *Sci. Rep.* **6**, 36031; doi: 10.1038/srep36031 (2016).

**Publisher’s note:** Springer Nature remains neutral with regard to jurisdictional claims in published maps and institutional affiliations.

## Supplementary Material

Supplementary Information

## Figures and Tables

**Table 1:** Summary of gas phase DMS (ppm) in the outflow of experimental flasks, and surface area and zooxanthellae cell density –normalised DMS concentrations. amol = 1 × 10^−18^ moles) Values represent mean (±total error) for each experimental stage (Stage I submersion, Stage II air exposure, Stage III re-submersion).

	Stage I Submersion	Stage II Air exposure	Stage III Re-submersion
**DMS**_**gas**_ **ppm**			
Acropora cf. horrida ^400^	0.1 ± 0.3	1.5 ± 1.1	0.1 ± 0.1
Porites cylindrica ^400^	0.04 ± 0.03	0.5 ± 0.1	0.02 ± 0.02
Seriatopora hystrix ^100^	0.3 ± 0.3	0.7 ± 0.1	0.1 ± 0.1
**DMS pmol/cm**^**2**^			
Acropora cf. horrida ^400^	53.7 ± 95.3	580.2 ± 456.6	30.5 ± 23.0
Porites cylindrica ^400^	1.3 ± 0.4	31.0 ± 8.8	1.9 ± 0.5
Seriatopora hystrix ^100^	7.3 ± 1.7	62.2 ± 18.1	17.4 ± 5.1
**DMS amol/cell**			
Acropora cf. horrida ^400^	24.2 ± 2.5	261.9 ± 21.1	10.2 ± 3.5
Porites cylindrica ^400^	0.6 ± 0.02	14.2 ± 0.4	1.3 ± 0.04
Seriatopora hystrix ^100^	1.0 ± 0.01	8.5 ± 0.01	5.3 ± 0.01
