# Supplementary material for: Air exposure of coral is a significant source of dimethylsulfide (DMS) to the atmosphere
Source: Sci Rep. 2016 Oct 31;6:36031. doi: 10.1038/srep36031 (PMC5086842; doi:10.1038/srep36031)
Supplement: Supplementary Information [file srep36031-s1.pdf]

Supporting Information

Air exposure of coral is a significant source of dimethylsulfide (DMS) to the atmosphere

Frances E. Hopkins[1], Thomas G. Bell[1,], Mingxi Yang[1], David J. Suggett[2,3], Michael Steinke[2]

[1] Plymouth Marine Laboratory, Prospect Place, Plymouth, PL1 3DH, United Kingdom.
[2] School of Biological Sciences, University of Essex, Wivenhoe Park, Colchester, CO4 3SQ, United Kingdom.
[3] Functional Plant Biology & Climate Change Research Cluster, University of Technology Sydney, PO Box 123, Broadway NSW 2007, Australia.

Online-only Supporting Information includes:

Extended Material and Methods

Supplementary figures: S1, S2

Supplementary tables: S1, S2, S3

References

Supporting Information

Extended Materials and Methods

Coral experimental facility. Independent colonies (genets) of three Indo-Pacific coral species (*Acropora* cf. *horrida, Porites cylindrica n = 4, Seriatopora hystrix n = 3*), originally from the same parent colony from the Indo-Pacific, were obtained from *Tropical Marine Centre Ltd.* (Chorleywood, UK). Each colony was used to generate between 1 and 25 (mean 11 ± 9) equal-sized nubbins (ramets), 2 - 5 cm tall. Using elongated forceps and pliers the nubbins were attached to 10 mm plastic PVC piping plugs with a non-toxic epoxy resin (Milliput® Standard). Ramets were followed throughout experimental design and selected from different genets to ensure biological replication for each treatment. All nubbins were distributed equally on holding racks receiving either 100 or 400 μmol photons m^{-2} s^{-1} respectively. It is worth noting that these light levels are significantly lower than maximum light levels in a natural reef environment, where noon irradiances can range from 700 – 1200 μmol photons m^{-2} s^{-1} [(1)].These two light intensities that were determined to be sub-saturating (100 μmol photons m^{-2} s^{-1}) and saturating (400 μmol photons m^{-2} s^{-1}) for calcification based on previous experiments[1].

All nubbins were kept on a 12:12 light: dark cycle within an acclimation tank maintained at 26 ± 1 °C [1]. Acclimation tanks were supplied with Tropic Marin® PRO REEF salt-based seawater supplemented with $NaHCO_3$ and $CaCl_2$ and circulated via a common biological sump of Fijian live rock (*Tropical Marine Centre* Ltd., Chorleywood, UK). Inorganic nutrient concentrations monitored every two days were undetectable throughout [1]. Alkalinity, determined from a Titrino titrator (*Metrohm*, Buckingham, UK), remained constant at 2.7 ± 0.2 μmol kg^{-1}. Nubbins were used for experimentation after 8-10 weeks of acclimation to these conditions, by which stage all were 3-8cm tall.

Determination of atmospheric DMS by API-CIMS. DMS_{gas} in the outflow of the coral flasks were determined using:

$$DMS_{gas} = \frac{Sig_{63}}{Sig_{66}} \cdot \frac{Flow_{std}}{Flow_{coral}} \cdot Conc_{66}$$

where $Flow_{std}$ and $Flow_{coral}$ are gas flow rates of the D3-DMS standard (5 mL min^{-1}) and the flow out of the coral incubation vessels (~60 mL min^{-1}), and $Conc_{66}$ is the D3-DMS gas standard mixing ratio (9.68 ppm, confirmed using an Eco Scientific DMS permeation tube and associated weight loss record). Sig_{63} and Sig_{66} represent the background-corrected API-CIMS signal (pA) for (protonated) DMS (m/z = 63) and D3-DMS (m/z = 66) respectively. Interference in Sig_{63} from the D3-DMS cylinder was ~1% of Sig_{66} (equivalent to ~0.008 ppm DMS_{gas} at 5 mL min^{-1}). During replicate flask experiments, DMS concentrations from each flask were averaged over the 5 min measurement period time (mean ± SE). For detailed information of the construction, theory of operation and general application of API-CIMS see Saltzman *et al.* (2009).[2]

Determination of atmospheric DMS by GC-FPD. Quantification of DMS concentrations in air samples was performed as described previously [3]. Briefly, headspace samples were used to calibrate the GC, following the determination of the Henry's Law constant for DMS in 0.5 M NaOH at 30°C (k_H^{cc} = the dimensionless ratio between the aqueous-phase concentration c_a of DMS and its gas-phase concentration c_g = 5.585). The gas chromatograph (GC-2010, Shimadzu, Milton-Keynes, UK) was operated isothermally at 40 °C with a 30m×0.53 mm×5 µm HP-1 capillary column (Agilent, Wokingham, UK). Injector and detector temperatures were set to 200 and 250 °C, respectively. The carrier gas (He) was supplied at 8.48 mL min^{-1} (linear velocity of 60 cm s^{-1}) and air and hydrogen supplied as flame gases at 70 and 60 mL min^{-1}. Under these conditions, DMS had an average retention time of 4.27 min.

Determination of seawater DMS, DMSP (total) and dissolved DMSO. Water samples were taken with gas-tight glass syringes through a sampling port in the lid of the flasks, and immediately processed for the determination of DMS, total DMSP (DMSPt) and dissolved DMSO (DMSOd) in the seawater media. For DMS, 5 mL of pre-filtered seawater media was purged and analysed by gas chromatography –flame photometric detection (GC-FPD) similarly to Vogt et a. (2008) [4]. For DMSPt in the seawater media, 7 mL of seawater was pipetted into an 8 mL glass vial, and the sample was fixed by addition of 35 µL of 50 % H_2SO_4 [5,6]. DMSPt analyses were performed at PML within 1 month of sample collection. DMSPt samples were hydrolysed with the addition of 10 M NaOH (0.5 - 1 mL) 4 – 6 h before analysis. Samples (0.5 – 2 mL) were withdrawn from the vial and purged and analysed by gas chromatography – pulsed flame photometric detection (GC-PFPD)[6,7].

For DMSOd, 2 mL of pre-filtered and pre-purged seawater was pipetted into new polypropylene vials, and acidified with 10 % HCl to give a final concentration of 0.05 M. Vials were sealed with Parafilm and stored at 4°C until analysis. Analysis was performed following established methods [8,9]. First, dissolved DMSP was removed by hydrolysing the sample with 300 µL 10 M NaOH to cleave all DMSPd to DMS, and 1-2 mL of sample purged at 60 mL min^{-1} for 5 min. Next, a pellet (~0.1 g) of $NaBH_4$ was added to the purge tower to reduce DMSOd to DMS. A gentle flow rate of 20 mL min^{-1} was applied to the sample to facilitate the reaction, whilst cryogenically trapping the DMS. The resultant alkaline solution was then re-acidified by addition of 200 µL 25 % HCl to the sample, and purged for a further 10 min at 60 ml min^{-1}. The pre-concentrated DMS was quantified by GC-PFPD as described above. Background DMSO was detected in the aquarium seawater. Measurements made in 'no coral' flasks (n = 9) during experiments gave a mean DMSOd concentration of 222.3 ± 123.9 (SD) nmol L^{-1}.

Determination of total DMSP content of coral. Total DMSP content of corals (tissue and skeleton) was determined in sub-nubbins (approximately 10% of nubbin) from the original experimental nubbins. Upon completion of the experiments, sub-nubbins were collected and placed in 10 mL 0.5 M NaOH in a 20 mL glass serum vial, and immediately crimp sealed. For analysis, samples were incubated at 30°C for 24 h, and then microliter volumes (3 – 40 µL) were withdrawn from each vial using a gas tight Hamilton syringe, followed by direct injection into the same GC system described above. For these measurements the GC oven was operated at 120 °C and the carrier gas flow was set to 10.56 mL min^{-1} (linear velocity of 80 cm s^{-1}) to give an approximate DMS retention time of 1 min [3]. DMSP content is normalised by the coral surface area (CSA) (Table S1). The mean (± SD) surface area (SA) of the *A.* cf. *horrida* nubbins was 41.9±27.8 cm^2. *P. cylindrica* and *S. hystrix* nubbins had SA (± SE) of 30.9±12.8 cm^2 and 28.3±11.6 cm^2 respectively (Table S3).

Determination of DMS concentration in coral mucus and mucus ropes. One colony each of *P. cylindrica* and *A.* cf. *horrida* were removed from the aquarium and exposed to the air for 15 minutes. After 15 minutes, known volumes of mucus that had collected on the surface of the corals were taken with a pipette and transferred to 4.92 mL headspace vials, and made up to 3 mL with seawater from the aquarium (samples of seawater were used as a control). Mucus ropes that formed upon re-submersion of *A.* cf. *horrida* were also collected and handled in the same way. Vials were vortexed for 1 min before a 200 µL headspace sample was withdrawn and injected directly into the injector of the GC-FPD [3]. The detection limit of DMS in a 200 µL headspace sample was ≤50 nM (the lowest concentration used in headspace calibrations) [3]. Note, these are not the same specimens used in the main experiments, but were sourced from the same supplier and kept under the same conditions in the same aquarium. Thus the data may not be fully representative of the experimental findings.

Additional coral variables. Additional variables were collected at the end of experimentation from all fragments. A Waterpik was used to remove a small area of tissue from each nubbin [10]; this area was then quantified using ImageJ. Tissue slurries produced were homogenised in a known volume of filtered seawater and a small aliquot removed for cell counts via a haemocytometer. The remaining volume was filtered through Glass Fibre Filters (*Whatman*) and immediately extracted in 5 mL of methanol for 24h at 4°C for subsequent chlorophyll *a* quantification [11]. Each nubbin was processed for buoyant weight and surface area (SA) using the paraffin wax technique [12], and surface area measurements were used to normalised all DMS, DMSP and DMSO data. Chlorophyll *a* and zooxanthellae density data is summarised in Table S3.

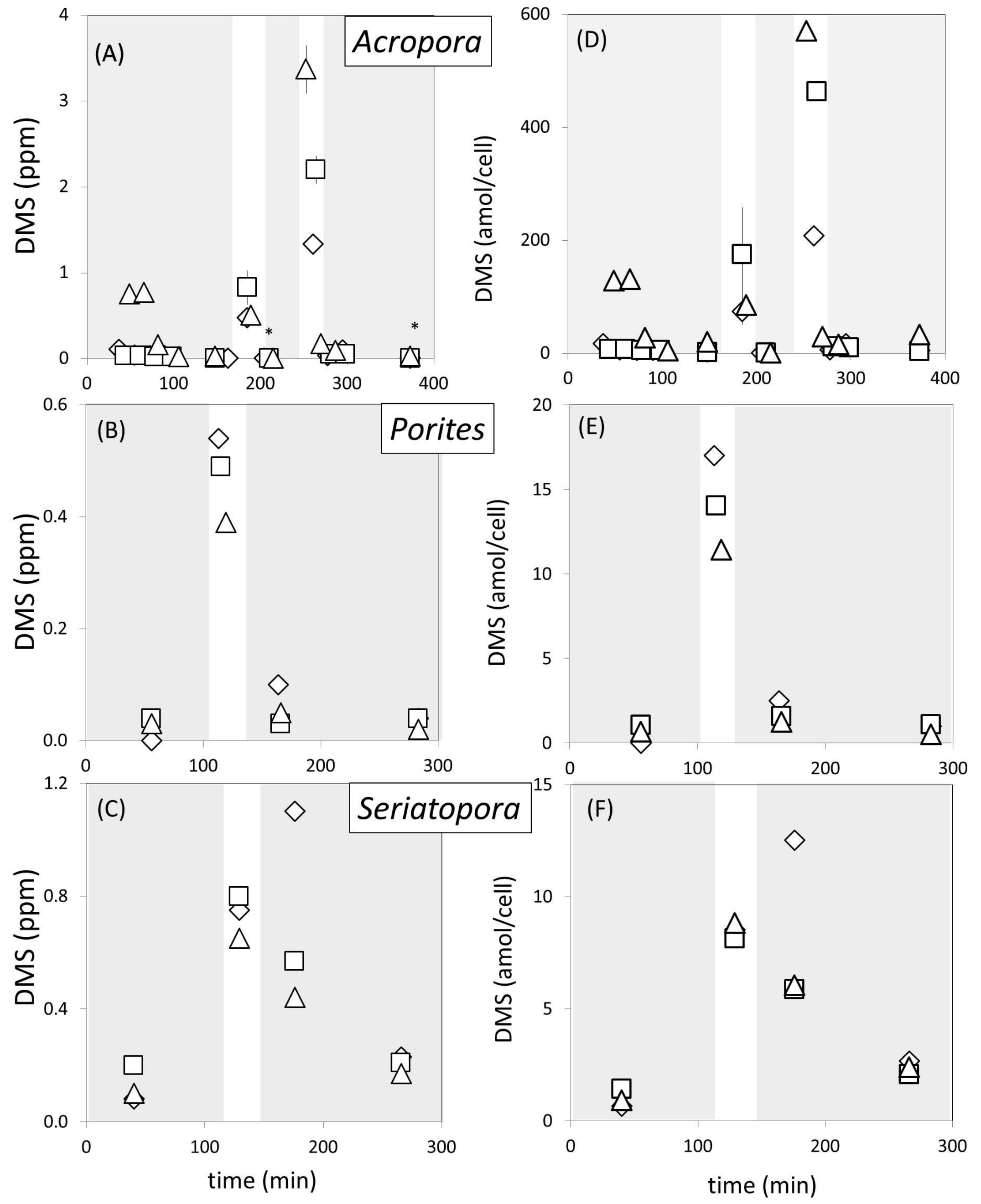

Figure S1. DMS_{gas} concentrations (ppm) in the outflow from triplicate coral experimental flasks (A – C) and DMS_{gas} normalised to zooxanthellae cell number (amol/cell) (D – F) before, during and after air exposure. Grey shaded areas = coral submersed (Stage I and III), white areas = coral exposed to air (Stage II). *Acropora* cf. *horrida*[400] (A, D), *Porites cylindrica*[400] (B, E) and *Seriatopora hystrix*[100] (C, F). Experiments were run in triplicate and replicate flasks are distinguished by different symbols (Flask 1 = diamonds, Flask 2 = squares, Flask 3 = triangles). Error bars on A and D represent the total measurement error which includes error associated with 5 min-averaged 0.25 Hz API-CIMS measurements (see Methods), coral surface area measurements and flow rate of culture aeration. Superscript numbers = light acclimation levels in μmol m^{-2} s^{-1}. * = discrete measurements made by GC-FPD.

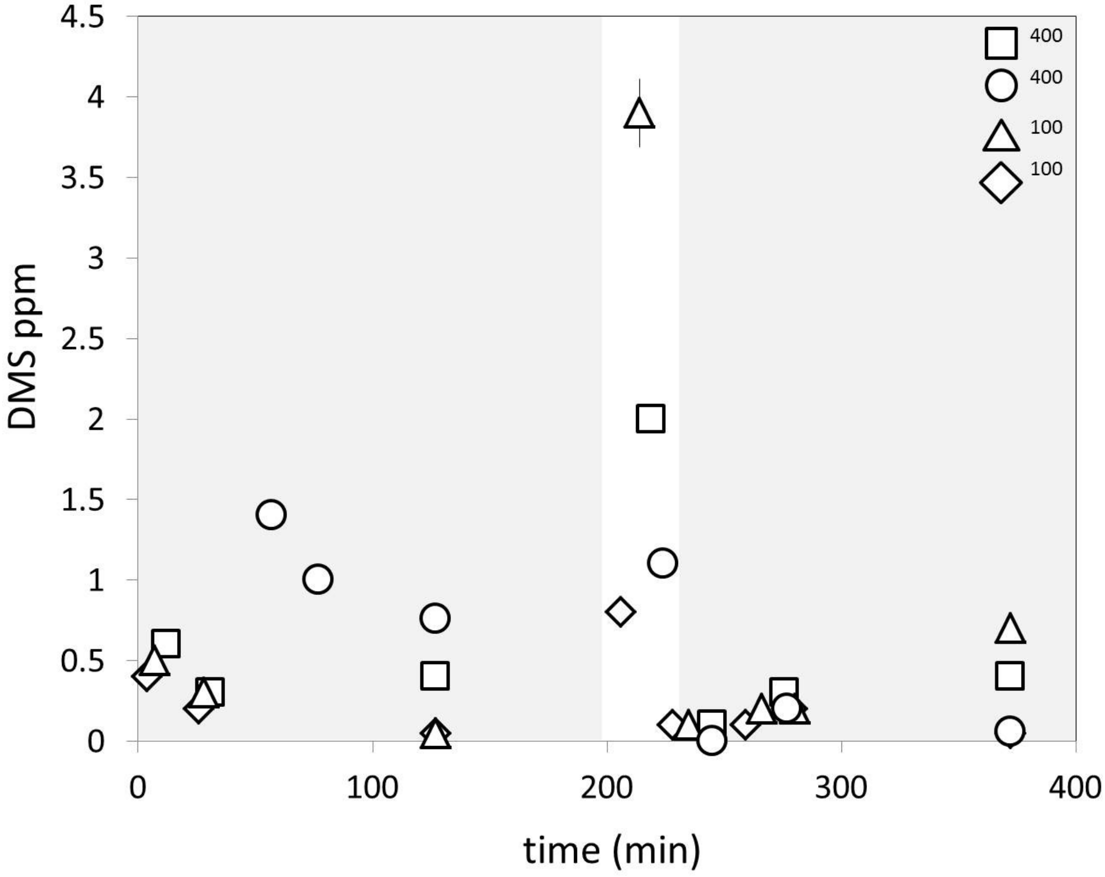

Figure S2. DMS_{gas} in the outflow from four experimental flasks holding nubbins (*n* = 3) of *A.* cf. *horrida*, before, during and after air exposure, and in the dark. Grey shaded areas = coral submersed (Stage I and III), white areas = coral exposed to air (Stage II). Symbols represent the data from four separate flasks. Flasks represented by squares and circles held coral nubbins that had been acclimated to 400 µmol photons m^{-2} s^{-1}, whilst flasks represented by triangles and diamonds held coral nubbins that had been acclimated to 100 µmol photons m^{-2} s^{-2}. Error bars (generally smaller than symbol size) represent the standard error on 5 min-averaged 0.25 Hz API-CIMS measurements (see Methods).

Supplementary Tables

Table S1. Summary of DMSP content normalised to surface area (nmol cm^{-2}) and zooxanthellate cell counts (fmol cell^{-1}) of experimental coral nubbins. Light acclimation level indicated by ^{100} (100 µmol photons m^{-2} s^{-1}) and ^{400} (400 µmol photons m^{-2} s^{-1}).

Species	DMSP nmol cm^{-2}	SD	SE	n	DMSP fmol cell^{-1}	SD	SE	n
Acropora cf. *horrida*^{100}	910.2	628.9	162.4	15	410.4	378.5	101.2	14
Acropora cf. *horrida*^{400}	1618.7	1333.9	444.6	9	1054.6	956.9	338.3	8
Porites cylindrica^{400}	79.7	71.8	21.7	11	41.5	45.6	14.4	10
Seriatopora hystrix^{100}	65.4	17.0	4.9	12	10.1	4.0	1.2	11
Seriatopora hystrix^{400}	38.1	22.3	9.1	6	12.1	2.9	1.3	5

Table S2. Summary of photophysiological data from short term emersion experiments using three tropical coral species. *Fq'/Fm'* = operating efficiency of PSII (dimensionless), τ′ = minimum electron turnover time (µs), σ′= effective absorption cross section (nm^2). Values are means (*SD*) from triplicate experiments performed in parallel to experiments for DMS production. Abbreviations: Stage I = submersion, Stage II = air exposure, Stage III =- re-submersion. ^{100} and ^{400} indicate light acclimation levels, 100 µmol m^{-2} s^{-1}, or 400 µmol m^{-2} s^{-1}. Experiments with *A.* cf. *horrida* were performed under both actinic light and dark conditions (^{d} = dark experiments).

Mean (*SD)*	*Fq'/Fm'*			τ′ (µs)			σ′ (nm^2)		
	Stage I	*Stage II*	*Stage III*	*Stage I*	*Stage II*	*Stage III*	*Stage I*	*Stage II*	*Stage III*
Acropora cf.. *horrida*^{400}	0.37 (0.02)	0.36 (0.01)	0.37 (0.02)	422.8 (4.2)	468.6 (11.0)	421.4 (3.1)	1.77 (0.01)	1.70 (0.03)	1.73 (0.01)
Acropora cf. *horrida*^{100}	0.34 (0.03)	0.25 (0.04)	0.33 (0.007)	426.8 (6.2)	495.8 (6.7)	420.0 (2.6)	2.12 (0.03)	1.63 (0.06)	1.98 (0.01)
Acropora cf. *horrida*$^{400, d}$	0.40 (0.003)	0.39 (0.001)	0.38 (0.003)	405.9 (8.4)	407.4 (4.6)	383.1 (4.7)	1.87 (0.06)	1.96 (0.03)	2.09 (0.02)
Acropora cf. *horrida*$^{100, d}$	0.42 (0.004)	0.40 (0.001)	0.40 (0.001)	390.2 (8.3)	399.9 (4.4)	381.3 (4.1)	2.12 (0.06)	2.60 (0.04)	2.32 (0.02)
Seriatopora hystrix^{400}	0.31 (0.01)	0.31 (0.004)	0.32 (0.005)	501.6 (8.2)	572.7 (5.3)	519.6 (4.1)	1.51 (0.02)	1.09 (0.03)	1.50 (0.01)
Seriatopora hystrix^{100}	0.36 (0.02)	0.29 (0.02)	0.34 (0.008)	479.9 (7.3)	577.0 (11.2)	495.0 (4.6)	1.15 (0.02)	0.85 (0.02)	1.14 (0.01)
Porites cylindrica^{400}	0.31 (0.02)	0.29 (0.01)	0.30 (0.01)	449.8 (9.9)	509.9 (13.8)	463.9 (4.1)	1.42 (0.02)	1.05 (0.05)	1.18 (0.02)

Table S3. Summary of mean surface area (cm^2), mean surface area normalised chlorophyll a concentrations (mg/cm^2) and mean zooxanthellate density ($cells/cm^2$) of experimental coral nubbins used in air exposure experiments. ^{100} and ^{400} indicate light acclimation levels, 100 $\mu mol\ m^{-2}\ s^{-1}$, or 400 $\mu mol\ m^{-2}\ s^{-1}$.

Species	Mean surface area cm^2	SD	Mean Chl *a* mg/cm^2	SD	Mean Zooxanthellae $density/cm^2$	SD
Acropora c.f. horrida ^{100}	46.6	32.6	2.7	1.7	2.6×10^6	1.1×10^6
Acropora c.f. horrida ^{400}	30.4	18.3	2.8	1.5	2.3×10^6	9.7×10^5
Acropora c.f. horrida (all)	41.9	27.8	2.7	1.6	2.5×10^6	1.0×10^6
Porites cylindrica ^{400}	30.9	12.8	2.3	2.4	2.3×10^6	8.0×10^5
Seriatopora hystrix ^{100}	25.4	7.8	0.8	0.7	7.4×10^6	2.8×10^6
Seriatopora hystrix ^{400}	33.3	13.5	0.9	0.4	4.8×10^6	2.6×10^6
Seriatopora hystrix (all)	28.3	11.6	0.9	0.6	6.2×10^6	2.9×10^6

References

1 Suggett, D. J. *et al.* Light availability determines susceptibility of reef building corals to ocean acidification. *Coral Reefs* **32**, 327-337, doi:10.1007/s00338-012-0996-7 (2013).

2 Saltzman, E., De Bruyn, W., Lawler, M., Marandino, C. & McCormick, C. A chemical ionization mass spectrometer for continuous underway shipboard analysis of dimethylsulfide in near-surface seawater. *Ocean Science* **5**, 537-546 (2009).

3 Steinke, M., Brading, P., Kerrison, P., Warner, M. E. & Suggett, D. J. Concentrations of dimethylsulfoniopropionate and dimethyl sulfide are strain-specific in symbiotic dinoflagellates (*Symbiodinium* sp., Dinophyceae). *J. Phycol.* **47**, 775-783 (2011).

4 Vogt, M. *et al.* Laboratory inter-comparison of dissolved dimethyl sulphide (DMS) measurements using purge-and-trap and solid-phase microextraction techniques during a mesocosm experiment. *Marine Chemistry* **108**, 32-39, doi:10.1016/j.marchem.2007.10.001 (2008).

5 Kiene, R. P. & Slezak, D. Low dissolved DMSP concentrations in seawater revealed by small-volume gravity filtration and dialysis sampling *Limnology and Oceanography Methods* **4**, 80-95 (2006).

6 Hopkins, F. E. & Archer, S. D. Consistent increase in dimethyl sulfide (DMS) in response to high CO2 in five shipboard bioassays from contrasting NW European waters. *Biogeosciences* **11**, 4925-4940, doi:10.5194/bg-11-4925-2014 (2014).

7 Archer, S. D. *et al.* Contrasting responses of DMS and DMSP to ocean acidification in Arctic waters. *Biogeosciences* **10**, 1893-1908, doi:10.5194/bg-10-1893-2013 (2013).

8 Andreae, M. O. Determination of trace quantities of dimethylsulfoxide in aqueous solutions. *Analytical Chemistry* **52**, 150-153 (1980).

9 Simó, R., Grimalt, J. O. & Albaiges, J. Dissolved dimethylsulphide, dimethylsulphoniopropionate and dimethylsulphoxide in western Mediterranean waters. *Deep-Sea Research II* **44**, 929-950 (1997).

10 Johannes, R. E. & Wiebe, W. J. Method for determination of coral tissue biomass and composition. *Limnology and Oceanography*, 822-824 (1970).

11 Ritchie, R. J. Consistent sets of spectrophotometric chlorophyll equations for acetone, methanol and ethanol solvents. *Photosynthesis Research* **89**, 27-41 (2006).

12 Bucher, D. J., Harriott, V. J. & Roberts, L. G. Skeletal micro-density, porosity and bulk density of acroporid corals. *Journal of Experimental Marine Biology and Ecology* **228**, 117-136 (1998)
